# Brain Mapping as Helpful Tool in Brain Glioma Surgical Treatment—Toward the “Perfect Surgery”?

**DOI:** 10.3390/brainsci8110192

**Published:** 2018-10-26

**Authors:** Fabio Barone, Nicola Alberio, Domenico Gerardo Iacopino, Giuseppe Roberto Giammalva, Corrado D’Arrigo, Walter Tagnese, Francesca Graziano, Salvatore Cicero, Rosario Maugeri

**Affiliations:** 1 Unit of Neurosurgery, Hospital “Cannizzaro”, 95100 Catania, Italy; fbarone2009@alice.it (F.B.); n.alberio@tin.it (N.A.); c.darrigo@quipo.it (C.D.); cicerosalvatore@yahoo.it (S.C.); 2 Neurosurgical Clinic, AOUP “Paolo Giaccone”, Post Graduate Residency Program in Neurologic Surgery, Department of Experimental Biomedicine and Clinical Neurosciences, School of Medicine, University of Palermo, 90127 Palermo, Italy; gerardo.iacopino@gmail.com (D.G.I.); franeurosurgery@libero.it (F.G.); rosario.maugeri1977@gmail.com (R.M.); 3 Intensive Care Unit, Hospital “Cannizzaro”, 95100 Catania, Italy; wtagnese@gmail.com

**Keywords:** awake surgery, asleep surgery, high grade glioma, low grade glioma, brain mapping, brain tumour, extent of resection

## Abstract

Gliomas are the most common primary malignant brain tumours in adults, representing nearly 80%, with poor prognosis in their high-grade forms. Several variables positively affect the prognosis of patients with high-grade glioma: young age, tumour location, radiological features, recurrence, and the opportunity to perform post-operative adjuvant therapy. Low-grade gliomas are slow-growing brain neoplasms of adolescence and young-adulthood, preferentially involving functional areas, particularly the eloquent ones. It has been demonstrated that early surgery and higher extent rate ensure overall longer survival time regardless of tumour grading, but nowadays, functional preservation that is as complete as possible is imperative. To achieve the best surgical results, along with the best functional results, intraoperative mapping and monitoring of brain functions, as well as different anaesthesiology protocols for awake surgery are nowadays being widely adopted. We report on our experience at our institution with 28 patients affected by malignant brain tumours who underwent brain mapping-aided surgical resection of neoplasm: 20 patients underwent awake surgical resection and 8 patients underwent asleep surgical resection. An analysis of the results and a review of the literature has been performed.

## 1. Introduction

Gliomas still represent a considerable challenge for neurosurgeons. Significant progress in disease control and in the management of recurrence have been achieved by medical and surgical therapy. As regards surgical management, it has been demonstrated that maximal resection of neoplasm has the greatest importance in order to gain better outcomes and survival rates [1,2,3].

Progress in neurosciences has provided a new representation of central nervous system (CNS) functions. The former organizations of CNS, comprising eloquent and non-eloquent areas, has gradually given way to a hodotopic view of the brain, where parallel streams of information come from multiple cortical areas, which are dynamically linked and modulated by interactive and distributed circuits [4].

According to these findings, higher neurological functions are the results of complex serial and parallel networks made of multiple neural areas. This is a multimodal scenario in which non-eloquent areas may have a role in processing and mediating these interactions in order to allow the expression of high brain functions, such as cognition and emotion [4].

Until now, only eloquent areas have generally been taken into account among the cortical regions closer to the surgical field during the preoperative evaluation of brain pathological processes [4]. However, a comprehensive evaluation of brain functions, from the sensory-motor ones to the higher cognitive ones, is required for adequate operative planning in the case of brain neoplasm such as low-grade glioma.

Low-grade gliomas are slow-growing brain neoplasms of adolescence and young-adulthood, which preferentially involve functional areas, particularly the eloquent ones. The molecular pathogenesis of these neoplasms is as yet poorly understood; they likely arise ex nihilo and grow, causing subtle neurological deficits [5,6,7,8,9]. According to the hodotopic model of the brain, grade II gliomas (LGGs) not only affect perilesional brain with their growing pattern, but they may also affect the connectivity of the whole brain [10].

LGGs often have an onset in which subtle neurological deficits, seizures, headache and personality change are exhibited [9,11,12]. In LGG patients, larger lesions are associated with higher risk of malignant transformation and shorter survival time [12]. Therefore, surgery is aimed to improve survival time and it represents the first therapeutic option for LGG, according to the current European Guidelines [13,14]. 

It has been demonstrated that early surgery and higher extent rate ensure overall longer survival time, but nowadays, preservation of brain functions that is as complete as possible is imperative [15]. In facts, LGGs often affect young patients with minor neurological deficits; therefore, surgery is advocated not only to treat the neoplasm but also to preserve patient’s functional independence [13].

To achieve the best surgical results, along with the best functional results, intraoperative mapping and monitoring of brain functions and different anaesthesiology protocol for awake surgery are nowadays widely adopted [12].

## 2. Materials and Methods

At our institution, 86 patients affected by brain neoplasm were evaluated during the last three years. Among these, 28 patients underwent brain mapping-aided surgical resection of neoplasm: 20 patients underwent awake surgical resection and 8 patients underwent asleep surgical resection.

Inclusion criteria for awake surgical resection were: age >18 years and <50 years, medium-high education, no preoperative motor/language deficit, dominant hemisphere and suggestive neuroimaging for critical language area involvement; exclusion criteria for awake surgery were: suggestive neuroimaging of pure motor area lesions, radiological evidence of “mass effect”, clinical signs of intracranial hypertension, dysmorphic oro-pharyngeal access (evaluated by the Mallampati score), other co-morbidity (heart diseases, respiratory diseases, metabolic failure), obesity, psychiatric disorders, and history of drug-resistant seizures; whereas elective criteria for asleep surgery were: age >50 years, suggestive neuroimaging of pure motor area lesions, mandibular hypoplasia, short and hypomobile neck, co-presence of slight motor deficit.

At our institution, every patient had undergone pre-operative morphological MRI and clinical evaluation since the first visit in order to suggest or exclude brain mapping-aided surgical resection. Every candidate for brain mapping-aided surgery underwent neurosurgical and anaesthesiological evaluation and complete pre-operative imaging by morphological Magnetic Resonance imaging (MRI), MRI-spectroscopy and brain positron emission tomography-computed tomography (PET-CT) scan with 11C-metionine in order to obtain a precise pre-operative evaluation. During the neurosurgical evaluation, every patient was screened for handedness through the Edinburgh Inventory. Then, exclusion and inclusion criteria for awake or asleep surgery were evaluated in order to plan the proper surgical procedure.

Several colloquies were performed with patients and their family in order to make them more confident with the surgical procedures. Before the surgical procedure, cognitive functions of each patient were assessed and patients were investigated about the possible maximum tolerated neurological sequelae according to their work, life-style and expectations.

## 3. Results

In our clinical series, we enrolled 28 patients—15 males and 13 females—whose mean age was 45 years (31 years–75 years, SD 13.3 years). Among the awake surgery patients, 11 patients suffered from LGG and 9 patients suffered from high grade glioma (HGG). Glioma was located in the temporal dominant lobe in 10 patients, in the frontal dominant lobe in 6 patients, in temporal-insular region in 3 patients and the inferior parietal lobule in one patient. As regards asleep surgery, 6 patients with peri-central HGG and 2 patients with non-dominant temporal-insular LGG underwent asleep surgical resection. In the awake surgery group, gross total resection (GTR) was obtained in 15 of 20 patients (9 HGG patients and 6 LGG patients) and subtotal resection (STR) was obtained in 5 of 20 patients. In the asleep surgery group, GTR was obtained in 2 of 8 patients and subtotal resection was obtained in 6 patients. Mean operative time was 4 h, 9 min (3 h, 15 min–5 h, 15 min).

As regards adverse events, in the awake surgery group 9 of 20 patients reported an immediate post-operative phonemic dysphasia, which was associated with a semantic dysphasia in 4 of them. All of these patients reported a complete relief from dysphasia within three months after proper rehabilitative interventions. In the asleep surgery group, 5 of 8 patients complained of an immediate post-surgical motor-deficit; two of them reported complete relief within ten days, three of them reported complete relief within three months, and a sole patient reported a permanent motor deficit. Our results are summarized in Table 1.

Common anaesthesiology protocols were adopted in case of general anaesthesia. The use of curare was avoided, and the use of anaesthetic gas was limited in order to support brain blood flow autoregulation during the procedure and to ensure a proper recording of motor-evoked potentials [16]. During awake surgery procedures, the anaesthesia was initially performed by the use of midazolam and remifentanil and a laryngeal mask. Local anaesthesia of the scalp was performed by the administration of mepivacain 0.2% and adrenalin 1/200,000 according to Regensburg protocol. A wide craniotomy was usually performed in order to expose frontal, temporal and parietal lobes. The local nervous block of dura mater was performed by the administration of local anaesthetics along the middle meningeal artery, then durotomy was performed. 

In our surgical protocol, during general anaesthesia, brain mapping was performed through the use of Ojemann’s stimulator with bipolar electrodes. This generates 60 Hz biphasic waves every 4 s, with a single pulse width of 1 ms. Stimulation starts ad 3 mA, with subsequent increments of 0.5 mA. We paid attention not to stimulate more than once the same cortical area in order to avoid afterdischarges. Moreover, we did not stimulate over 12 mA. Intraoperative seizures and afterdischarges were promptly treated by local cortical irrigation with cold Ringer lactate solution.

During the procedure, tumour resection was usually performed with the aid of ultrasonic suction (CUSA system). As regard intraoperative evaluation of language functions in the dominant hemisphere, we initially adopted 1.5–2 mA direct current, with subsequent increments of 0.5–1 mA until a functional response was evoked or to the maximum current limit of 6 mA. As usual, language mapping was initially performed by the evaluation of speech arrest, in order to localize the language motor area, then patients were asked to perform a naming task during surgical resection [17,18]. As regards the non-dominant hemisphere, brain mapping during awake surgery was finalized to identify the superior longitudinal fascicle in order to avoid the neglect syndrome.

## 4. Discussion

LGGs (grade II gliomas) are mostly considered aggressive neoplasms due to their slow mean growth rate, to their infiltrative growth in brain parenchyma and white matter tracts and their tendency to malignant progression [12]. Despite resistant seizures induced by large tumours, LGGs often induce subtle neurological symptoms that require a specific neuropsychological assessment in order to be detected [12]. Due to the high prevalence among young people without gross neurological deficits, LGG requires a fine and sharp surgical technique in order to treat drug-resistant seizure, extend survival time, delay the time of malignant progression and improve neurological functions [12]. In this scenario, the goal of the surgery should be to maximize the resection of tumour ant to minimize the neurological morbidity. This is defined as “onco-functional balance” and should be the primary objective of brain mapping aided-surgery for LGGs [10,12].

### 4.1. Brain Mapping Techniques

Brain mapping consist of several pre-operative and intra-operative techniques, which were developed to facilitate a wider extent of neoplasm while preserving brain functional areas [11]. These techniques are widely used to perform surgical resection of high- and low-grade gliomas involving eloquent brain areas [11].

### 4.2. Anatomic and Functional Pre-Operative Imaging

To correctly assess a patient’s neurological functions and to plan the surgical treatment in the safest way, a pre-operative anatomical and functional evaluation is mandatory. This evaluation is performed through standard pre-operative MRI. This provides the anatomical basis for the further functional studies, such as functional MRI (fMRI) and diffusion tensor imaging-fibre tracking (DTI-FT) [11,12,19].

Anatomical MRI is usually performed through T1-weighted, T2-weighted, fluid-attenuated inversion recovery (FLAIR), T1-contrast images and volumetric sequences. In this way MRI is used to provide as much as possible information about the tumour location. These imaging studies can be integrated with MR spectroscopy and MR perfusion sequences, which provide metabolic information about the lesion [11]. Also, single photon emission computed tomography (SPECT) and positron emission tomography (PET) can be used to obtain metabolic information about the tumour and its biological behaviour [11]. All these data can be stored and elaborated on neuronavigation systems, which provide a virtual model of the brain in order to plan the surgery and to guide the surgeon during the procedure. 

Functional MRI (fMRI) provides information about cortical areas, which are activated in response to motor or language tasks. It is useful to correlate the tumour location with the functional anatomy of the cortex when an involvement of motor or language areas is suspected [11]. fMRI is often integrated by DTI-FT, which depicts subcortical tracts through anisotropy of water diffusion in myelinated fibres. DTI-FT is used to evaluate the relationship between tumour mass, subcortical tracts and surrounding enema, allowing the distinction between unchanged, dislocated, infiltrated and interrupted tracts [19]. It has been shown that combined used of fMRI and DTI-FT together with brain mapping techniques and the use of neuronavitagion systems enhances the accuracy in identification of functional areas and eloquent tracts. Moreover, it has been demonstrated that these techniques ensure a greater preservation of neurological functions, decrease surgical time and risk of seizures in a series of 230 patients who undergone surgery for LGG (176) and HGG (154) [19].

### 4.3. Intraoperative Functional and Anatomical Evaluation

As regards intraoperative functional evaluation, a board of neurophysiological tests are currently adopted. Electroencephalography and electrocorticography are frequently used to evaluate cortical activity. Electroencephalography is used during the early stage of the surgery, through subdermal needle electrodes; electrocorticography is used in the later stage of surgery when the brain cortex has been exposed. They are used continuously in order to monitor brain basal electrical activity, to detect afterdischarge during electrical stimulation, and to monitor the occurrence of seizures during surgical resection [11]. Motor functions are evaluated by electromyography and motor-evoked potentials, which are capable of providing information on the integrity of the motor pathways and indicate impending brain ischemia both in asleep and awake patients [11,12]. To test on-line motor, language and cognitive functions during surgery, direct electrical stimulation (DES) is used for cortical and subcortical mapping. Once the stimulation parameters have been set, DES is applied through an electrode to verify the function of an area before it is resected. The failure during intraoperative continuous neuropsychiological assessment because of direct electrical stimulation on certain areas informs that those areas are involved in certain functional pathway; thus, a neurological deficit would arise from their resection [11,12]. In our practice, we apply 60 Hz biphasic waves every 4 s, with a single pulse width of 1 ms. In asleep patients, DES starts at 3 mA, with subsequent increments of 0.5 mA, until we detect an evoked instrumental response, and do not exceed the limit of 12 mA; in awake patients we usually start at 1 mA and use growing current until functional response is detected, without exceeding 6 mA as a limit.

As regards the intraoperative anatomical definition of the neoplasm, surgical resection is often aided by the use of intraoperative fluorescent markers, such has 5-aminolevulinic acid (5-ALA) and fluorescein sodium (FS). These markers are adopted to visually differentiate tumour from normal surrounding brain under a proper wavelength light and a proper fluorescence light filter on the operative microscope. These tools are used to enhance the identification of tumour borders and to better define the surgical resection. Moreover, it has been proven that the use of fluorescent markers together with intraoperative brain mapping tools allows a more radical resection with an extent of up to 100% within the functional limits of the surrounding brain [20,21].

### 4.4. Brain Mapping-Aided Surgical Resection

All these intraoperative functional evaluations are adopted together in different settings to perform motor, language, and visuospatial mapping according to the tumour location and to the functional anatomy of the brain [12]. It has been shown that the risk of post-operative motor or language deficits is 72.8% when the resection is kept 5 mm far from eloquent cortical areas and 65.4% when the resection is pushed to the boundaries of subcortical functional tract; if no subcortical tract is evidenced during the resection by brain mapping techniques, the risk of post-operative deficit is 3–5%. Even if post-operative deficits are transient and most of them disappear within 1 month, a 3.8% risk of permanent deficit has been shown in cases of subcortical resection involving functional tracts [12]. Usually, resection is continued until subcortical structures are found, then it has to be stopped in order to avoid neurological injury. In our daily practice, autologous fibrin glue and haemostatic agents are adopted to ensure a safer resection, limiting the risk of post-surgical bleeding within the surgical field and favouring also dural sealing [22,23,24,25]. When tumour resection is performed according to these techniques, the risk of postoperative long-term neurological impairment is very low. It has been shown that almost all patients return to work 1 month after the surgery [12].

### 4.5. Neurological and Cognitive Evaluation

Neuropsychological impairments have been encountered in more than 90% LGG patients [11]. Since tumour growth may affect social, behavioural, emotive and cognitive functions, a complete evaluation of brain function is mandatory in order to preserve them [11]. Moreover, tumour excision may provoke several neurological deficits, as consequence of the damage of eloquent areas or interference in hodotopic network, even if the resection is guided by DES [10,26]. Thus, precise pre-operative planning is mandatory to tailor the surgical strategy to the single case and to the possible post-operative neurological sequalae that the patient would tolerate [10,26]. During awake surgery, intraoperative neurocognitive assessment is performed through visuospatial, emotional, memory, planning, learning, attention and behavioural tasks, according to the tumour location and to the functional areas involved in the resection [10]. Language mapping is usually performed through the evaluation of spontaneous speech, object naming, counting, reading and writing. As regards sensory-motor functions, they are evaluated through evoked potentials and motor stimulation during asleep surgery, whereas during awake surgery, it is possible to assess somatosensory, visual, vestibular functions and spatial cognition [10].

Considering the necessity of assessing brain functions not only on the basis of anatomical localization of eloquent areas, pre-operative neuropsychological assessment and the patient’s life characteristics should guide the selection of intra-operative tasks, according to the concept of “onco-functional balance” [10,27].

### 4.6. Surgical Resection and Onco-Functional Balance

The aim of LGG surgery is to obtain a good impact on the natural history of the neoplasm, to reach the best onco-functional balance and to preserve patient’s quality of life. Since the anatomy alone is not sufficient in those cases, resection has to be performed according to the boundaries of brain functions, and the surgery should be tailored according to the neural network of the brain [3,10,14,26].

It has been widely demonstrated that the extent of resection (EOR) is a reliable predictor of overall survival (OS) in LGG patients [28]. In a series of 2016 LGG, Smith et al. demonstrated that a complete tumour resection is associated with 8-years SO in 98% of patients [29]; moreover, it has been confirmed that gross total resection is independently associated with higher OS [30]. Secondly, OS in LGG patients appears to be directly related to EOR [31] and it has been demonstrated that incomplete resection results in 4.9 times greater risk of death than complete resection [32]. As regards the association between EOR and SO, an at least 80% EOR is a significant predictor of OS, whereas 90% EOR ensure a significantly better OS [29].

As regards the onco-functional balance, brain mapping plays a fundamental role in LGG surgery. In a meta-analysis involving 8091 glioma patients it has been demonstrated that brain mapping-aided surgery for glioma resection presents a lower rate of severe neurological sequelae (less than 3.5%) and a wider extent of resection, even involving more frequently eloquent areas [33,34,35].

Even if our series is limited, in the asleep surgery group, we obtained GTR less frequently and encountered a higher rate of permanent neurological deficit compared to the awake surgery group. However, according to our experience, awake surgery entails a higher rate of immediate post-surgical neurological deficits, even if they are prompt to recovery.

According to Gil-Robles et al., LGG resection may be safely conducted even beyond the security margins around eloquent structures. In fact, in a series of 162 LGG patients, it was demonstrated that wide resection until the limits of eloquent areas and functional structures is possible through the aid of direct electrical stimulation. Surprisingly, the lack of safe margins did not increase the rate of permanent neurological deficits, which has been shown to be about 2% of all cases [34]. Brain plasticity allows the surgeon to safely overcome the ancient anatomical boundaries of the eloquent areas. Thanks to brain plasticity, surgeons may also plan a “multi-staged” surgery of gliomas; in this way, it is possible to resect during a second surgery parts of the brain not allowed to be resected during the first operation, since they were functionally involved.

As regards functional assessment in LGG involving eloquent areas, in a series of 11 patients, Duffau et al. demonstrated that resection of such LGG is safely possible. In particular, a transient worsening of neurological functions was noted in 63% of patients. Despite this, all patients improved after a proper rehabilitation, and all the patients totally recovered [36]. In another series of 25 patients, Mandonnet et al. reported complete resection in 14 patients, subtotal resection in 8 patients, partial resection in 3 patients with different grade gliomas by the use of brain mapping aided surgery. Post-operative neurological deficits were observed in 16% of patients and 80% of working patients resumed their employment [37]. As regards awake surgery for LGG, in a series of 67 glioma patients undergoing awake craniotomy, Chacko et al. reported that the awake procedure was well tolerated by all the patients. Post-operative neurological deficits were reported in 13.4% of patients, and no improvement was reported during follow-up in only 5.9% of them [38]. In that series, the authors reported a lower risk of post-operative deficit if surgical resection was stopped at the boundary between the tumour and the positive mapping cortex and white matter [38].

As regards oncological result, Bello et al. demonstrated that the use of brain mapping techniques can increase the number of patients undergoing surgical resection from 11% to 81%; moreover, an increased rate of total and sub-total resection from 11% to 69.8% was achieved with the application of brain mapping techniques [11,12].

At present, the nature and biology of gliomas do not allow the regression of the neoplasm and the complete healing of the patient. Therefore, multimodal treatment of gliomas should include the latest medical and surgical tools in order to optimize the surgical resection and to maintain patient’s quality of life as far as possible. In this way, it can be possible to avoid a premature decline of the patient and the unavoidable social, familiar and psychological marginalization [39]. 

## 5. Conclusions

Significant progress in the treatment of gliomas has been obtained by medical and surgical therapy, and nowadays, maximal resection of neoplasm still has the greatest importance in terms of gaining better outcomes and survival rates.

To achieve the best post-operative onco-functional balance, intraoperative brain mapping, along with anaesthesiology protocols for awake surgery, are nowadays advisable.

Brain mapping consists of several pre-operative and intra-operative techniques that are adopted to evaluate brain functional areas and to facilitate the wider extent of neoplasm. It has been proven that brain mapping allows a safe and optimized resection with lower rate of postoperative neurological deficits, a greater preservation of the patient’s neurological function, and a better onco-functional balance.

## 6. Case Illustration

### “Awake” Resection of a Right Fronto-Opercular Diffuse Low-Grade Glioma in a Left-Handed Patient

**Figure 1:**
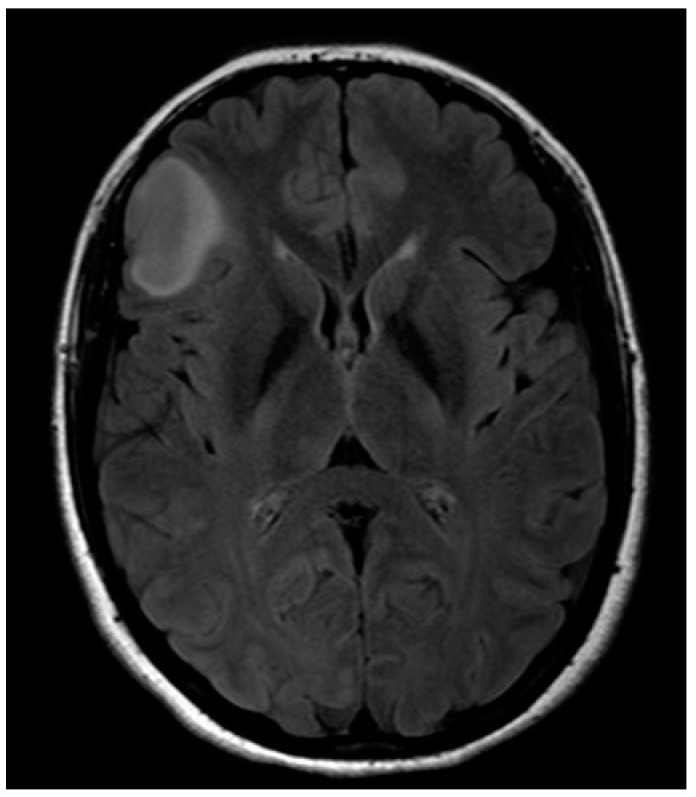
Anatomical MRI scan showing right fronto-opercular diffuse low-grade glioma.

A 32-year-old left-handed female patient was referred to our department with an MR scan showing a right fronto-opercular diffuse low-grade glioma and performed as a diagnostic work-up for headache (Figure 1). Left-handedness was confirmed with Edinburgh’s inventory. She had no preoperative language disorders.

**Figure 2:**
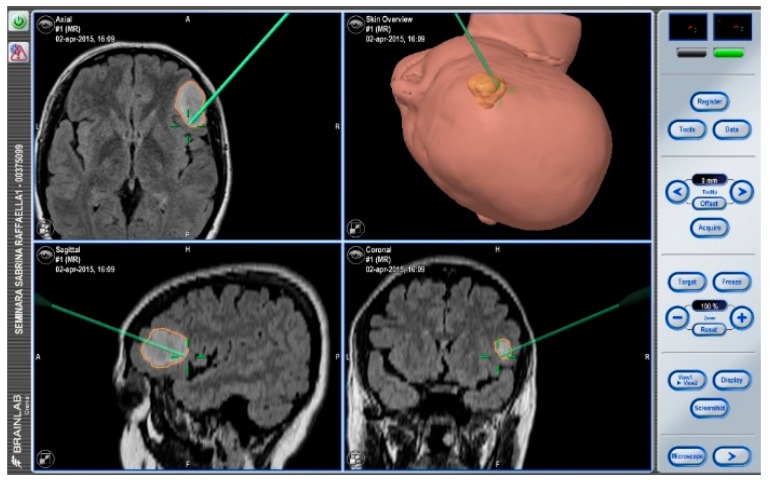
Neuronavigation system adopted at our Institution.

**Figure 3:**
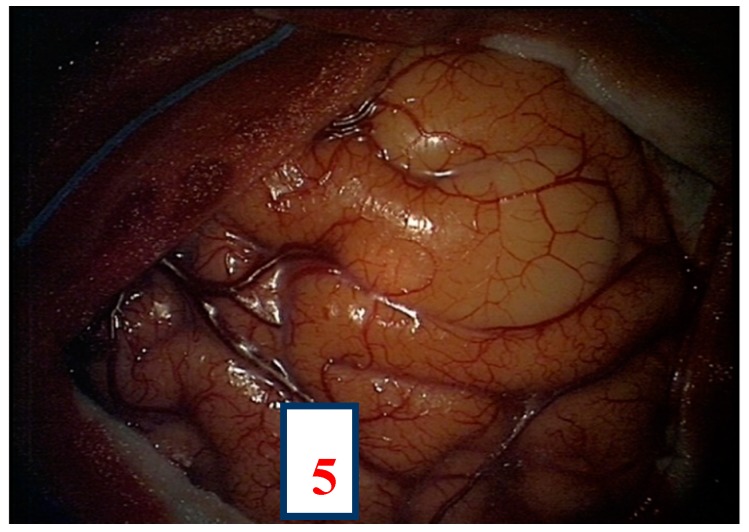
Intra-operative picture showing Broca’s area (number 5) identified by direct electrical stimulation.

**Figure 4:**
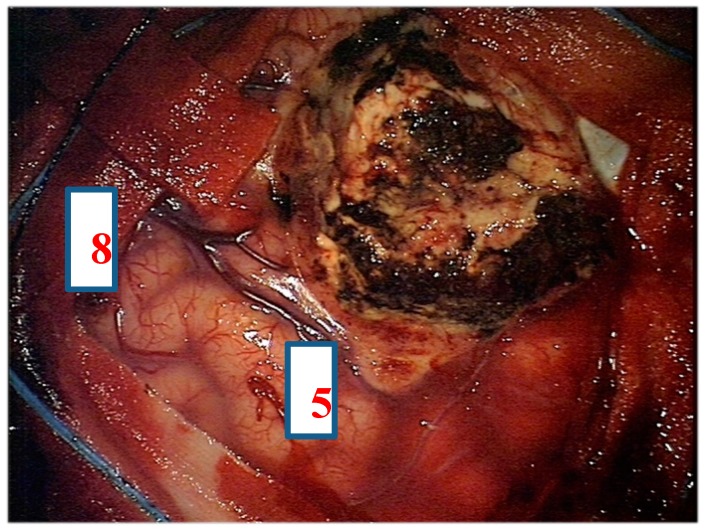
Intra-operative picture showing Broca’s area (number 5) and inferior fronto-occipital fascicle (IFOF) (number 8) identified by direct electrical stimulation.

After navigation-guided identification of the tumour boundaries, an “awake” resection was performed and intraoperative identification of Broca’s area (tag 5) with “speech arrest” and inferior fronto-occipital fascicle (IFOF) with transient fonemic paraphasia was reached (tag 8) Figure 2, Figure 3 and Figure 4. Resection was then stopped. 

**Figure 5:**
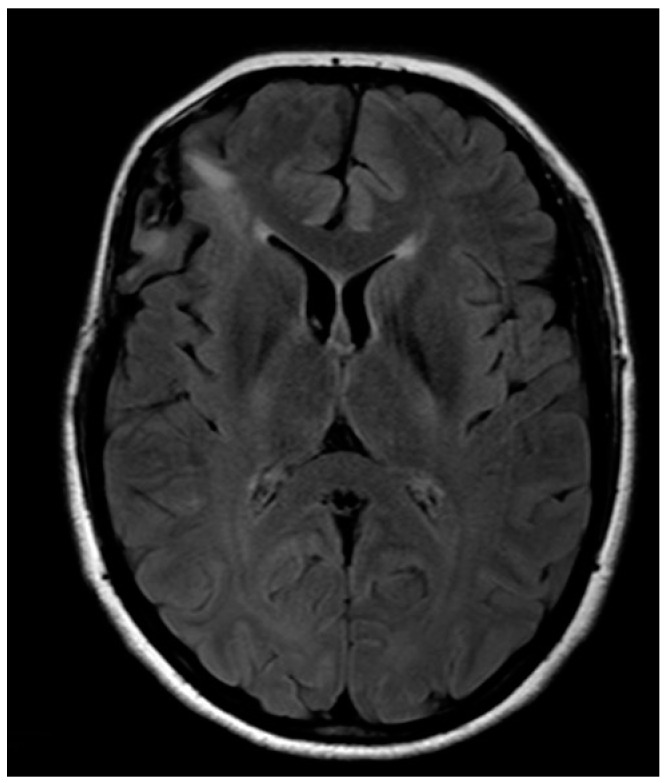
Post-operative anatomical MRI.

Histology provided evidence for isocitrate dehydrogenase 1(IDH1)-mutated, ATP-Dependent Helicase (ATRX)-wild diffuse low-grade astrocytoma. Postoperative MR showed a subtotal removal with residual disease where IFOF was intraoperatively identified (Figure 5). No adjuvant therapy was then proposed, and the patient is currently fully working. If evidence of residual disease progression is observed, a new surgery will be proposed, delaying eventual adjuvant therapy as much as possible.

This may be considered an example case of the “multi-staged” approach to dealing with diffuse low-grade gliomas, making it possible to get control of the disease as much as possible, postponing chemotherapy or radiotherapy as far as possible and reserving them for when surgery can no longer be performed.

## Figures and Tables

**Table 1:** Patients’ demographics and characteristics.

Case No.	Sex/Year	Primary Neoplasm/Location	Procedure	Pre-Operative Investigation	Intraoperative Mapping Techniques	Duration of the Surgery	Extent of Resection	Post-Operative Sequelae
1	M/31 years	Low grade glioma (LGG) (astrocytoma)/frontal	Awake	Anatomical MRI, magnetic resonance spectroscopy (MRS), ^11^C-Met-PET	DES, MEPs, motor/language task	4 h–20 min	GTR	Phonemic dysphasia
2	F/22 years	LGG (astrocytoma)/temp-insul	Awake	“	“	4 h–30 min	STR	Phonemic/semantic dysphasia
3	M/43 years	LGG (oligoastro)/temp-insul	Awake	“	“	3 h–30 min	STR	-
4	M/33 years	LGG (astrocytoma)/temporal	Awake	“	DES, language test	4 h	GTR	Phonemic dysphasia
5	M/49 years	HGG (GBM)/temporal	Awake	“	“	4 h–45 min	GTR	-
6	F/34 years	LGG (astrocytoma)/temp-insul	Awake	“	DES, MEPs, motor/language task	5 h	STR	Phonemic/semantic dysphasia
7	F/39 years	HGG (astro III)/frontal	Awake	“	“	3 h–50 min	GTR	-
8	F/48 years	HGG (GBM)/temporal	Awake	“	DES, language test	4 h	GTR	Phonemic dysphasia
9	M/27 years	LGG (oligodendro)/frontal	Awake	“	DES, MEPs, motor/language task	4 h–20 min	GTR	-
10	M/42 years	HGG (epend III)/IPL	Awake	“	DES, language test	5 h–15 min	GTR	Phonemic/semantic dysphasia
11	F/43 years	LGG (astrocytoma)/frontal	Awake	“	DES, MEPs, motor/language task	3 h–45 min	STR	Phonemic dysphasia
12	F/46 years	LGG (astrocytoma)/SMA	Awake	“	“	4 h–15 min	GTR	-
13	F/28 years	HGG (oligodendro III)/frontal	Awake	“	“	3 h–50 min	GTR	Phonemic dysphasia
14	F/34 years	LGG (astrocytoma)/temporal	Awake	“	DES, language test	4 h–20 min	GTR	Phonemic/semantic dysphasia
15	M/41 years	LGG (astrocytoma)/temporal	Awake	“	“	4 h–30 min	STR	Phonemic dysphasia
16	M/49 years	HGG (GBM)/temporal	Awake	“	“	4 h–20 min	GTR	-
17	M/48 years	HGG (GBM)/temporal	Awake	“	“	3 h–20 min	GTR	Phonemic dysphasia
18	F/30 years	LGG (oligodendro)/temporal	Awake	“	“	4 h–15 min	GTR	Phonemic dysphasia
19	M/48 years	HGG (GBM)/temporal	Awake	“	“	4 h	GTR	-
20	F/49 years	HGG (GBM)/temporal	Awake	“	“	4 h–30 min	GTR	Phonemic dysphasia
21	M/57 years	HGG (GBM)/perirolandic	Asleep	“	DES, MEPs, SSEPs	3 h–20 min	STR	-
22	F/74 years	HGG (GBM)/perirolandic	Asleep	“	“	3 h–15 min	STR	Motor deficit <10 days
23	M/55 years	LGG (astrocytoma)/temp-ins nd	Asleep	“	“	4 h–20 min	STR	-
24	M/41 years	LGG (astrocytoma)/temp-ins nd	Asleep	“	“	4 h–30 min	STR	-
25	M/64 years	HGG (GMB)/perirolandic	Asleep	“	“	4 h–30 min	STR	Motor deficit <10 days
26	F/62 years	HGG (GBM)/perirolandic	Asleep	“	“	4 h	GTR	Motor deficit <3 months
27	M/75 years	HGG (GBM)/perirolandic	Asleep	“	“	4 h	STR	Permanent motor deficit
28	F/60 years	HGG (GBM)/perirolandic	Asleep	“	“	3 h–50 min	GTR	Motor deficit <3 months

Demographics and characteristics of 30 patient undergone brain mapping-aided surgery. GTR: gross total resection. STR: Sub-total resection. MRI: Magnetic resonance imaging. MRS: magnetic resonance spectroscopy. ^11^C-Met-PET: L-methyl-11C-methionine positron emission tomography. DES: direct electrical stimulation. MEPs: motor evoked potentials. SSEPs: somatosensory evoked potentials.
